# Bioaffinity Ultrafiltration Combined with HPLC-ESI-qTOF-MS/MS for Screening Potential Bioactive Components from the Stems of *Dendrobium fimbriatum* and In Silico Analysis

**DOI:** 10.3390/antiox13080918

**Published:** 2024-07-29

**Authors:** Yu-Hui Hsieh, Wu-Chang Chuang, Ming-Chung Lee, Yu-Hsin Fan, Nai-Kuei Huang, Jih-Jung Chen

**Affiliations:** 1 Biomedical Industry Ph.D. Program, School of Life Sciences, National Yang Ming Chiao Tung University, Taipei 112304, Taiwan; hsieh.ls10@nycu.edu.tw; 2 Brion Research Institute of Taiwan, New Taipei City 231030, Taiwan; 3 Sun Ten Pharmaceutical Co., Ltd., New Taipei City 231030, Taiwan; mileslee@sunten.com.tw; 4 Department of Pharmacy, School of Pharmaceutical Sciences, National Yang Ming Chiao Tung University, Taipei 112304, Taiwan; cindyfan.md12@nycu.edu.tw; 5 National Research Institute of Chinese Medicine, Ministry of Health and Welfare, Taipei 112304, Taiwan; 6 Ph.D. Program for Neural Regenerative Medicine, College of Medical Science and Technology, Taipei Medical University, Taipei 110301, Taiwan; 7 Graduate Institute of Medical Sciences, College of Medicine, Taipei Medical University, Taipei 110301, Taiwan; 8 Department of Medical Research, China Medical University Hospital, China Medical University, Taichung 404333, Taiwan; 9 Traditional Herbal Medicine Research Center, Taipei Medical University Hospital, Taipei 110301, Taiwan

**Keywords:** *Dendrobium fimbriatum*, different solvent extracts, anti-acetylcholinesterase activity, anti-inflammatory activity, antioxidant activity, bioaffinity ultrafiltration, molecular docking

## Abstract

*Dendrobium fimbriatum* is a perennial herb, and its stems are high-grade tea and nourishing medicinal materials. Various solvent extracts of *D. fimbriatum* were evaluated for their anti-inflammatory, anti-acetylcholinesterase (AChE), antioxidant, and anti-α-glucosidase properties. Acetone and EtOAc extracts showed significant antioxidant effects. Acetone, *n*-hexane, and EtOAc extracts revealed potent inhibition against α-glucosidase. EtOAc, *n*-hexane, and dichloromethane extracts displayed significant anti-AChE activity. Among the isolated constituents, gigantol, moscatin, and dendrophenol showed potent antioxidant activities in FRAP, DPPH, and ABTS radical scavenging tests. Moscatin (IC_50_ = 161.86 ± 16.45 μM) and dendrophenol (IC_50_ = 165.19 ± 13.25 μM) displayed more potent anti-AChE activity than chlorogenic acid (IC_50_ = 236.24 ± 15.85 μM, positive control). Dendrophenol (IC_50_ = 14.31 ± 3.17 μM) revealed more efficient anti-NO activity than quercetin (positive control, IC_50_ = 23.09 ± 1.43 μM). Analysis of AChE and iNOS inhibitory components was performed using molecular docking and/or the bioaffinity ultrafiltration method. In bioaffinity ultrafiltration, the binding affinity of compounds to the enzyme (acetylcholinesterase and inducible nitric oxide synthase) was determined using the enrichment factor (EF). Among the main components of the EtOAc extract from *D. fimbriatum* stem, moscatin, dendrophenol, gigantol, and batatasin III with acetylcholinesterase exhibited the highest binding affinities, with affinity values of 66.31%, 59.48%, 54.60%, and 31.87%, respectively. Moreover, the affinity capacity of the identified compounds with inducible nitric oxide synthase can be ranked as moscatin (88.99%) > dendrophenol (65.11%) > gigantol (44.84%) > batatasin III (27.18%). This research suggests that the bioactive extracts and components of *D. fimbriatum* stem could be studied further as hopeful candidates for the prevention or treatment of hyperglycemia, oxidative stress-related diseases, and nervous disorders.

## 1. Introduction

**Figure 1:**
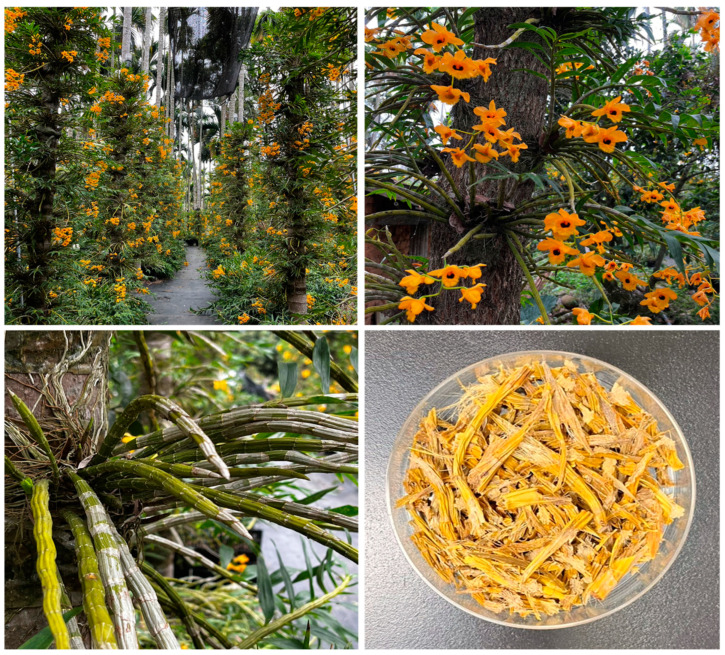
Dried stems of *Dendrobium fimbriatum* were used in this study and collected from Mingjian Township, Nantou County, Taiwan.

The dried stems of Dendrobium species have been used as high-grade tea and nourishing medicinal materials [1]. However, some of these species have not been produced on a large scale, resulting in high prices for Dendrobium tea and a lack of species-specific bioactivity studies [2]. Taiwan is famous for its high-quality orchid exports. In Taiwan, the warm and humid climate is very suitable for growing orchids [3]. *Dendrobium fimbriatum*, an Orchidaceae plant [4], is one of the Dendrobium species recorded in the *Chinese Pharmacopoeia*, with the functions of nourishing Yin, clearing heat, promoting saliva, and protecting the stomach [5]. Pharmacological research has demonstrated that Dendrobium extracts, which are rich in polysaccharides, alkaloids, benzyl compounds, and other bioactive substances [5,6,7], display antioxidative, anti-inflammatory, hypoglycemic, hepatoprotective, and hypolipidemic effects [8,9,10,11]. The stem of *Dendrobium fimbriatum* is a famous herbal medicine and a high-quality healthy food [12] that has been successfully cultivated in Nantou, Taiwan by Sun Ten Pharmaceutical Co., Ltd. A comparative evaluation of the active ingredient content of *D. fimbriatum* was conducted, and the results revealed that *D. fimbriatum* grown for three years had the highest active ingredient content. Therefore, the stems of three-year-grown *D. fimbriatum* (Figure 1) were used as research materials in this study.

Ultrafiltration mass spectrometry (UF-LC/MS) stands as a robust bioanalytical approach that combines affinity ultrafiltration with liquid chromatography–mass spectrometry (LC/MS) [13]. This innovative technique facilitates the swift screening and simultaneous identification of small active molecules binding to specific biological targets of interest [14]. By exploiting the affinities between these small molecular compounds and key enzyme protein targets, UF-LC/MS swiftly isolates and characterizes active components from intricate natural products [15]. Diverging from alternative analytical methods, affinity UF-LC/MS offers distinct advantages, including rapidity, sensitivity, and high throughput, rendering it especially well suited for the intricate extracts obtained from medicinal plants [16].

Today, with an aging global population, the prevalence of multiple chronic diseases is increasing, placing a burden on healthcare systems [17]. During aging, the metabolic system in the body declines, leading to obesity in the elderly, which conversely results in elevated levels of reactive oxygen species and chronic inflammation, resulting in oxidative damage to cells and tissues, including DNA, RNA, proteins, carbohydrates, and cell membrane lipids [18]. Thus, tissues undergoing these processes may develop insulin resistance and neurodegeneration, leading to diabetes and Alzheimer’s disease [19]. NO is a free radical involved in the regulation of many physiological processes, such as vascular relaxation, neurotransmission, platelet aggregation, and immune regulation. NO is produced by nitric oxide synthase (NOS) from molecular oxygen and L-arginine, yielding L-citrulline as a by-product [20]. In the presence of cytotoxins produced by inflammatory substances in the environment, the expression of inducible nitric oxide synthase (iNOS) increases, causing NO to be produced. Excessive NO can lead to inflammation [21]. Therefore, lowering ROS levels and suppressing inflammation in the body is crucial for preventing chronic diseases. In addition, the discovery of bioactive natural compounds targeting chronic disease-associated enzymes may also help alleviate chronic diseases [19]. Natural products have been a common source of health supplements for the past few decades [22]. Therefore, this article conducted a study on the herbal tea *D. fimbriatum* to evaluate its anti-inflammatory, antioxidant, anti-α-glucosidase, and anti-acetylcholinesterase effects, looking for a health supplement that can help promote health.

## 2. Materials and Methods

### 2.1. Chemicals and Reagent

Nitroblue tetrazolium (NBT), phenazine methosulphate (PMS), and 2,2-diphenyl-1-(2,4,6-trinitrophenyl) hydroxyl (DPPH) were procured from Tokyo Chemical Industry Co., Ltd. (Tokyo, Japan). Various chemicals were purchased from Sigma-Aldrich (St. Louis, MO, USA), which included chlorogenic acid, Trolox, sodium dodecyl sulfate (SDS), bovine serum albumin, 2,2′-azino-bis(3-ethylbenzothiazoline-6-sulfonic acid) (ABTS), gallic acid, Folin–Ciocalteu reagent, 5,5-dithio-bis-(2-nitrobenzoic acid) (DTNB), 2,4,6-tris(2-pyridyl)-s-triazine (TPTZ), EDTA, sodium bicarbonate, α-glucosidase, inducible nitric oxide synthase (iNOS), calcium chloride (CaCl_2_), calmodulin, magnesium chloride (MgCl_2_), flavine adenine dinucleotide (FAD), flavine-mononucleotide (FMN), β-nicotinamide adenine dinucleotide 2′-phosphate reduced tetrasodium salt hydrate (NADPH), glutathione, (6R)-5,6,7,8-tetrahydrobiopterin dihydrochloride (BH-4), HEPES, dithiothreitol (DTT), arginine, nitric oxide synthase (NOS), acetylcholinesterase, lipopolysaccharide, acetylcholine iodide, and dimethyl sulfoxide (DMSO). Potassium acetate, acarbose, sodium acetate, butylated hydroxytoluene (BHT), and nicotinamide adenine dinucleotide (NADH) were acquired from Acros Organics (Geel, Belgium). Ferric chloride (FeCl_3_), aluminum chloride (AlCl_3_), and *p*-nitro-phenyl-α-D-glucopyranoside (*p*-NPG) were obtained from Alfa Aesar (Lancashire, UK). Disodium hydrogen phosphate, sodium carbonate, potassium peroxydisulfate, potassium dihydrogen phosphate, and sodium dihydrogen phosphate were purchased from SHOWA Chemical Co., Ltd. (Chuo-ku, Japan). Quercetin was bought from MedChemExpress (Monmouth Junction, NJ, USA). Glycine was supplied by J.T. Baker (Phillipsburg, NJ, USA). TEMED and ammonium persulfate were acquired from Bio-Rad (Hercules, CA, USA). Tween20 was provided by Merck (Darmstadt, Germany).

### 2.2. Preparation of D. fimbriatum Extract

The stems of *D. fimbriatum* were planted and collected in three-year-grown plants from Mingjian Township, Nantou County, Taiwan, and were verified by Prof. J.-J. Chen. Some dried stems of *D. fimbriatum* were stored as voucher specimens in the Department of Pharmacy, NYCU. The stems of *D. fimbriatum* were air-dried and crushed. An amount of 20 g of dried stem powder was soaked in 100 mL of deionized water, ethanol, methanol, ethyl acetate, acetone, n-hexane, and dichloromethane, respectively. The mixture was sonicated for 1 h and allowed to stand at 25 °C for 3 days. The extract was filtered through Whatman No. 1 filter paper and concentrated at 37 °C. The 100 °C water extract was prepared by soaking 40 g of dried stems of *D. fimbriatum* in 1000 mL of deionized water for 30 min, and then boiling the mixture for 30 min until half of the liquid part remained. Then, the extract was filtered and concentrated at 37 °C. Each solvent extract was stored at −20 °C.

### 2.3. Preparation of Pure Components

**Figure 2:**
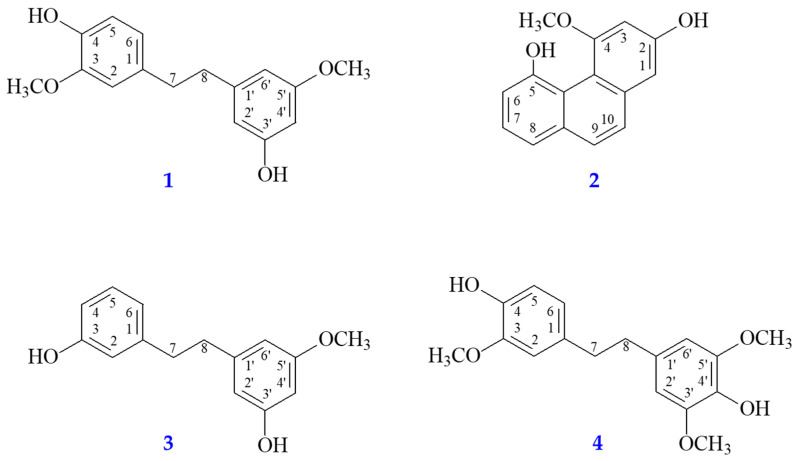
Chemical structures of gigantol (**1**), moscatin (**2**), batatasin III (**3**), and dendrophenol (**4**) from *D. fimbriatum*.

The dried stems of *D. fimbriatum* (1 kg) were crushed and extracted three times with EtOAc. The EtOAc extract was concentrated to obtain a dry extract (Fraction A, 7.09 g). Fraction A (7.09 g) was separated with column chromatography (CC) (300 g of silica gel, 70–230 mesh, *n*-hexane/acetone gradient) to acquire 15 fractions: A1–A15. Part (97 mg) of fraction A7 (97 mg) was separated by preparative TLC (silica gel; *n*-hexane/ethyl acetate, 2:3) to obtain batatasin III (8.83 mg, Rf = 0.80) and moscatin (11.78 mg, Rf = 0.51). Part (118 mg) of fraction A9 was separated with preparative TLC (silica gel; CH_2_Cl_2_/acetone, 17:3) to give dendrophenol (9.84 mg, Rf = 0.74) and gigantol (11.50 mg, Rf = 0.46). The isolated compounds gigantol, dendrophenol, batatasin III, and moscatin are shown in Figure 2.

### 2.4. Reverse-Phase HPLC

Reverse-phase HPLC analysis of *D. fimbriatum* was carried out with the method previously reported [23]. The samples were analyzed applying Waters^®^ e2695 Separations Module, Waters 2489 UV/Vis Detector, and Inertsil ODS-2 HPLC Column (5 μm, 150 × 4.6 mm). The mobile phase consisted of acetonitrile (A) and deionized water (B) using the following gradient program: 0–6 min, linear gradient from 14.7 to 16.2% A; 6–25 min, linear gradient from 16.2 to 60% A; 25–27 min, linear gradient from 60 to 100% A; 27–30 min, linear gradient maintained at 100% A; 30–32 min, linear gradient from 100 to 14.7% A; and 32–35 min, back to initial conditions at 14.7% A. The injection volume was 10 μL, the flow rate was 1.0 mL/min, and the analytes were monitored at 280 nm. Finally, HPLC analysis of all crude extracts and isolated compounds was performed.

### 2.5. Determination of Total Phenolic Content (TPC)

TPC was determined by the Folin–Ciocalteu procedure [24]. Each solvent extract was diluted to 100 μg/mL with deionized water. Gallic acid was used to create a standard curve. Then, 200 μL of each solvent extract and gallic acid were mixed with 200 μL of Folin–Ciocalteu (0.5 N, diluted with deionized water) in sealed tubes, and 400 μL of 20% Na_2_CO_3_ solution was added to each sealed tube. The optical density of each mixture was obtained at 750 nm after incubation for 40 min in the dark. TPC of the extracts was calculated using a gallic acid standard curve, and TPC of each solvent extract was displayed as mg of gallic acid equivalent (GAE) per g of extract.

### 2.6. Determination of Total Flavonoid Content (TFC)

TFC was measured by the AlCl_3_ colorimetry method [24]. The solvent extract was diluted to 100 μg/mL with MeOH. Quercetin was used to create a standard curve. An amount of 200 μL of the diluted solvent extract and quercetin solution was added to each sealed tube, and 100 μL of 10% AlCl_3_ and 100 μL of 0.1 mM CH_3_COOK were added to each sealed tube. The mixtures were incubated for 30 min. Finally, the optical density was measured at 415 nm. TFC of each solvent extract was calculated by applying a quercetin standard curve and indicated as mg quercetin equivalents (QCEs) per g of extract.

### 2.7. DPPH Radical Scavenging Activity

The DPPH radical scavenging effect was measured based on the reported method [25]. BHT was applied as a positive control. DPPH solution was prepared diluted by EtOH to reach the final concentration of 400 µM. Different concentrations (400, 200, 100, 50, and 25 µg/mL) of extracts or compounds (100 µL) were mixed with DPPH solution (100 µL) and incubated for 30 min at room temperature and in darkness, and the absorbance of the mixture was measured at 520 nm.

### 2.8. ABTS Radical Scavenging Activity

The ABTS cation radical scavenging effect was measured, as previously described by [25]. The stock of working solution was prepared mixing 28 mM ABTS solution and 9.6 mM potassium persulfate with double-distilled water (final concentration, 1/1, *v*/*v*) and leaving the mixture in the dark at 4 °C overnight (about 16 h). The working solution was diluted by EtOH to reach the absorbance of 0.70 ± 0.02 at 740 nm for further experiments. Different concentrations (400, 200, 100, 50, and 25 µg/mL) of extracts or compounds (10 µL) were mixed with the working solution (190 μL). The mixture was incubated at room temperature for 6 min, and the antioxidant activity of the mixture was determined by the decrease in absorbance measured at 740 nm.

### 2.9. Superoxide Radical Scavenging Activity

A superoxide radical scavenging assay was performed based on previously reported methods [25]. The superoxide radical was generated in 16 mM Tris-HCl buffer (pH 8.0) containing 50 μL of NBT (300 μM), 50 μL of PMS (120 μM), and 50 μL of different concentrations of the test sample. The reaction was initiated by adding 50 μL of NADH (468 μM) solution to the mixture. After incubating at room temperature for 5 min, the activity of samples was determined by the decrease in absorbance measured at 560 nm against the sample concentration.

### 2.10. Ferric Reducing Antioxidant Power (FRAP)

FRAP was measured by the method reported in the previous literature [25]. The working solution was mixed with acetate buffer (pH 3.6), ferric chloride solution (20 mM), and TPTZ solution (10 mM TPTZ in 40 mM HCl) at a proportion of 10:1:1, respectively, and freshly prepared before being used. A total of 900 μL of the working solution was warmed to 37 °C and then mixed with 100 μL of the diluted sample, blank or standard, in a microcentrifuge tube. The tubes were vortexed in a dry bath at 37 °C for 40 min. The absorbance was measured at 593 nm. The standard curve was linear between 0 and 100 mM Trolox. Results were expressed as mM TE/g dry weight. Additional dilution was needed if the FRAP value measured was over the linear range of the standard curve.

### 2.11. α-Glucosidase Inhibitory Activity Assay

To test the anti-α-glucosidase effect of each solvent extract and each component, a previously described method was used [24]. The positive control used in the experiment was acarbose. The α-glucosidase solution was diluted to 1 U/mL with 0.1 M sodium phosphate buffer (pH 6.8). Various concentrations of extract or compound (100 µL) and α-glucosidase solution (20 µL) were mixed. Subsequently, 0.53 mM *p*-NPG (380 µL) was added and incubated at 37 °C for 40 min. The reaction was stopped by adding 500 μL of 0.1 M Na_2_CO_3_ solution, and the absorbance was detected at 405 nm.

α-glucosidase inhibition (%) was counted as follows (A_sample_ and A_control_ represent the absorbance of the tested sample and control, respectively):

α-glucosidase inhibition (%) = (A_control_ − A_sample_)/A_control_ × 100%

### 2.12. Acetylcholinesterase Inhibitory Assay

The acetylcholinesterase inhibitory assay was carried out applying a method described in reference [26]. Briefly, 0.1 M sodium phosphate buffer (pH 8.0, 140 μL), DTNB (10 μL), sample (20 μL), and AChE solution (15 μL) were added to a 96-well microplate and incubated at room temperature for 10 min. Finally, the absorbance was detected with a spectrophotometer at 405 nm.

The acetylcholinesterase inhibitory effect was counted as follows (A_sample_ and A_control_ represent the absorbance of the tested sample and control, respectively):

Acetylcholinesterase inhibitory activity (%) = (A_control_ − A_sample_)/A_control_ × 100%

### 2.13. Bioaffinity Ultrafiltration Assay

The bioaffinity ultrafiltration assay was conducted using a previously described procedure [27,28,29]. First, inactive acetylcholinesterase was obtained by heating 100 μL of active enzyme (10 U/mL) in a dry bath at 100 °C for 10 min. The crude extract (5 mg/mL) was mixed with equal volumes (100 µL) of active and inactive acetylcholinesterase (10 U/mL), respectively. The reaction was conducted at 37 °C for 30 min, then centrifuged using a 14,000× *g* ultracentrifuge filter at 30 °C for 15 min. The filtrate was removed and 200 μL of Tris-HCl buffer was added to the ultracentrifuge filter and centrifuged at 14,000× *g* for 15 min at 30 °C. The previous step was repeated 3 times and the filtrate was removed. Then, 200 μL of methanol was added to the ultracentrifuge filter and centrifuged at 14,000× *g* for 15 min at 30 °C. The previous step was repeated 3 times and the filtrate was collected. The filtrate was concentrated and then dissolved in 50 μL MeOH for HPLC analysis. In addition, the processing of iNOS-ligand complexes was conducted following previously reported methods [28,30], while inducible nitric oxide synthase bioaffinity ultrafiltration assays were performed, as detailed above.

The binding affinity of compounds and enzymes using enrichment factor (EF) was assessed, and was counted as follows: A_1_, A_2_, and A_0_ represent the peak areas of picked compounds acquired from the extracts incubated with activated, inactivated, and no acetylcholinesterase, respectively.

EF (%) = (A_1_ − A_2_)/A_0_ × 100%

### 2.14. Cell Culture

In this experiment, the RAW 264.7 macrophage cell line was used. The cells were stored at −196 °C in liquid nitrogen. After thawing, the cells were cultured in DMEM at 37 °C and 5% CO_2_ supplemented with 10% FBS, 2 μM L-glutamine, 1 mM sodium pyruvate, 100 μg/mL streptomycin, and 100 U/mL penicillin.

### 2.15. Nitric Oxide (NO) Inhibition Assay

NO inhibition assay was slightly modified from the reported method [26]. The day before the experiment, RAW 264.7 cells were seeded into 96-well plates with 2 × 10^4^ cells per well. The cells were pretreated with the sample solution, and then incubated at 37 °C for 1 h. After incubation, the cells were treated with 100 ng/mL of LPS to induce an inflammatory response. After incubation at 37 °C for 20 h, 50 μL of the supernatant was transferred from each well to another 96-well plate and mixed with an equal volume of Griess reagent. Finally, the absorbance was acquired at 550 nm after 20 min of incubation.

### 2.16. Cytotoxicity Assay

Cytotoxicity was tested using MTT, as previously reported [26,31].

### 2.17. Western Blotting

Western blotting was conducted with the previous procedure [24]. Cells were treated with different concentrations of compounds and 100 ng/mL of LPS for a day. After rinsing cells with cold PBS, proteins were harvested with radioimmunoprecipitation assay buffer (RIPA buffer) and stored at −20 °C for succeeding procedure. Proteins were separated on a 12.5% SDS-polyacrylamide gel and then electrophoretically transferred to a PVDF membrane. The membrane was soaked in 2% bovine serum albumin (BSA) blocking buffer for 2 h, then washed 2–3 times with TBST. Proteins were treated by particular primary antibodies and immersed overnight. The following day, PVDF membranes were washed with TBST to remove residual primary antibodies, and then immersed with secondary antibodies for 2 h. Then, enhanced chemiluminescence solution was coated on the membrane and visualized using an ImageQuant LAS 4000 Mini Biomolecular Imager (GE Healthcare, Marlborough, MA, USA). Finally, we quantified band density by ImageJ software 1.53a (BioTechniques, New York, NY, USA).

### 2.18. Molecular Modeling Docking Study

Molecular docking models of compounds and enzymes were performed, applying previously reported methods [25,26]. The affinity of bioactive compounds and target enzymes was calculated using Discovery Studio 2019 software. The 3D structures of the ligands were created in ChemDraw Ultra 12.0. The crystal structures of AChE (PDB ID: 1C2B) and iNOS (PDB ID: 1M9T) were downloaded from the Protein Data Bank. Then, hydrogen atoms were added to the protein molecules. Subsequently, structures of protein and compound were imported into CDocker for molecular docking analysis, and 10 different docking positions were calculated and ranked by binding energy. Optimal docking poses were visualized and analyzed to find binding amino acid residues.

### 2.19. Statistical Analysis

All data are shown as mean ± SEM. Statistical analysis was performed applying Student’s *t*-test. Probabilities of 0.05 or less were thought statistically significant. All the assays were carried out at least three times.

## 3. Results and Discussion

### 3.1. TPC, TFC, and Yields of Different Solvent Extracts

The TPC, TFC, and extraction yields of various solvent extracts of *D. fimbriatum* stems were determined, and the results are indicated in Table 1. Calibration curves for the calculations related to TPC and TFC are provided in Figures S1 and S2. Significant differences exist in the yields of various solvent extracts, ranging from 0.30 ± 0.08% (*n*-hexane extract) to 19.70 ± 1.64% (100 °C water extract). Among the various solvent extracts, the yield of the water extract at 100 °C was the highest, which may be due to the highly polar components in the stems of *D. fimbriatum*. Acetone extract (34.48 ± 1.50 mg/g) and ethyl acetate extract (33.92 ± 0.75 mg/g) exhibited the highest TPC, whereas *n*-hexane extract (1.67 ± 0.27 mg/g) showed the lowest TPC. The TFC of various solvents decreased with the increase in polarity of extraction solvent, and the TFC of *n*-hexane extract (51.53 ± 1.88 mg/g) was the highest, followed by dichloromethane extract (49.69 ± 1.96 mg/g), ethyl acetate extract (44.05 ± 0.65 mg/g), acetone extract (34.20 ± 3.43 mg/g), and ethanol extract (3.46 ± 0.26 mg/g).

### 3.2. DPPH Radical Scavenging Effect

As displayed in Table 2 and Table 3, the scavenging effect of each solvent extract and isolated compound against DPPH radicals was evaluated. BHT was applied as a positive control. Ethyl acetate extract (SC_50_ = 53.08 ± 8.37 μg/mL) showed the most potent DPPH radical scavenging effect. Acetone and dichloromethane extracts also exhibited significant DPPH radical scavenging effects, with SC_50_ values of 55.89 ± 7.68 and 88.44 ± 13.19 μg/mL, respectively. Among the isolated compounds, dendrophenol (SC_50_ = 11.64 ± 0.77 μM) exhibited the most potent DPPH radical scavenging effect, followed by moscatin and gigantol, with SC_50_ values of 26.66 ± 1.63 and 35.18 ± 1.34 μM, respectively. The DPPH radical scavenging effects of dendrophenol, moscatin, and gigantol were all stronger than the positive control BHT (SC_50_ = 90.77 ± 5.65 μM).

### 3.3. ABTS Cation Radical Scavenging Effect

The antioxidant capacity of each solvent extract and isolated compound was investigated by measuring their scavenging activity against ABTS anion radicals. BHT was applied as a positive control. The results are displayed in Table 2 and Table 3. The EtOAc extract showed the strongest free radical scavenging activity against ABTS cations, with SC_50_ values of 57.62 ± 5.38 μg/mL, followed by acetone and CH_2_Cl_2_ extracts, with SC_50_ values of 59.78 ± 5.26 and 108.47 ± 9.03 μg/mL, respectively. All isolated components exhibited stronger ABTS radical scavenging effect than BHT (SC_50_ = 53.72 ± 1.48 μM), among which dendrophenol displayed the strongest activity (SC_50_ = 7.64 ± 0.63 μM), followed by moscatin (SC_50_ = 10.34 ± 1.92 μM), gigantol (SC_50_ = 12.25 ± 0.54 μM), and batatasin III (SC_50_ = 30.14 ± 1.70 μM).

### 3.4. Superoxide Radical Scavenging Effect

The superoxide radical scavenging effect of different extracts and isolated compounds was evaluated. Due to the poor solubility of BHT in the assay buffer, cynaroside, a potent antioxidant previously reported [32], was applied as a positive control. As indicated in Table 2 and Table 3, neither various solvent extracts (IC_50_ > 400 μg/mL) nor isolated compounds (IC_50_ > 400 μM) showed significant superoxide radical scavenging effects.

### 3.5. Ferric Reducing Antioxidant Power

The antioxidant activity of solvent extracts and isolated compounds was assessed for the reduction of ferric ions to ferrous ions and was shown as equivalents of Trolox per gram of tested sample. The results are displayed in Table 2 and Table 3. Among all solvent extracts, the EtOAc extract exhibited the best ferric reducing effect (TE = 882.70 ± 7.26 mM/g), followed by acetone and CH_2_Cl_2_ extracts (TE = 861.19 ± 11.31 and 562.26 ± 14.21 mM/g, respectively), while *n*-hexane, water, and hot water extracts showed low ferric reducing effects. On the other hand, among the isolated compounds, dendrophenol possessed the highest ferric reducing activity (TE = 1686.53 ± 16.61 mM/g), followed by gigantol, moscatin, and batatasin III (TE = 1483.20 ± 63.49, 1396.40 ± 57.03, and 559.67 ± 51.49 mM/g, respectively).

As displayed in Table 2, the ethyl acetate extract exhibited most potent antioxidant activity in the DPPH, ABTS, and FRAP assays. HPLC analysis of ethyl acetate extract showed that the main ingredients were gigantol, moscatin, and dendrophenol. These ingredients also showed potent antioxidant activity. Therefore, we inferred that the potent antioxidant activity of the ethyl acetate extract was due to the presence of these three main active components: gigantol, moscatin, and dendrophenol.

The 1,2-diphenylethane derivatives, dendrophenol (**4**), exhibited more potent antioxidant activity than their analogues, gigantol (**1**), batatasin III (**3**), and the phenanthrenoid moscatin (**2**) in the DPPH, ABTS, and FRAP assays (Table 3). Among the 1,2-diphenylethane analogues (Table 3), gigantol (**1**) and dendrophenol (**4**) (with 4-hydroxy-3-methoxyphenethyl moiety) exhibited more potent antioxidant activity than batatasin III (**3**) (with 3-hydroxyphenethyl moiety) in the DPPH, ABTS, and FRAP assays.

### 3.6. α-Glucosidase Inhibitory Effect

As displayed in Table 4 and Table 5, all solvent extracts and isolated components were evaluated for α-glucosidase inhibitory effects. Among all extracts, the acetone extract showed the best inhibitory activity against α-glucosidase (IC_50_ value = 51.62 ± 0.84 μg/mL), followed by ethyl acetate, *n*-hexane, and dichloromethane extracts, with IC_50_ values of 62.02 ± 0.21, 91.43 ± 2.87, and 294.09 ± 8.61 μg/mL, respectively. The above extracts displayed more effective inhibition against α-glucosidase than acarbose (positive control, IC_50_ = 316.30 ± 11.15 μg/mL). Among the isolated compounds, gigantol displayed the most potent anti-α-glucosidase activity (IC_50_ = 469.72 ± 28.51 μM), followed by moscatin, dendrophenol, and batatasin III (IC_50_ = 478.46 ± 33.43, 608.37 ± 48.16, and 723.21 ± 53.54 μM, respectively). Gigantol and moscatin exhibited more effective anti-α-glucosidase activity than acarbose (IC_50_ = 490.00 ± 17.23 μM).

### 3.7. Acetylcholinesterase (AChE) Inhibitory Activity

All solvent extracts and isolated compounds were assayed for anti-AChE effects, and the results are indicated in Table 4 and Table 5. Among all extracts, EtOAc extract (IC_50_ = 11.59 ± 1.52 μg/mL) and *n*-hexane extract (IC_50_ = 13.77 ± 4.09 μg/mL) possessed the strongest inhibitory activity against AChE. Dichloromethane, ethanol, and 100 °C water extracts also showed strong inhibitory activities against AChE, with IC_50_ values of 31.89 ± 6.89, 51.96 ± 4.92, and 75.87 ± 7.91 μg/mL, respectively. All above extracts displayed stronger inhibitory effects than chlorogenic acid (positive control, IC_50_ = 75.99 ± 6.21 μg/mL). Among the isolated compounds, moscatin (IC_50_ = 161.86 ± 16.45 μM) possessed the best inhibitory effect against AChE, followed by dendrophenol, gigantol, and batatasin III (IC_50_ = 165.19 ± 13.25, 176.05 ± 19.08, and 258.29 ± 18.52 μM, respectively). Moscatin and dendrophenol exhibited more potent anti-AChE effects than chlorogenic acid (IC_50_ = 236.24 ± 15.85 μM).

As displayed in Table 4, EtOAc extract exhibited the most potent anti-AChE activity. HPLC analysis of EtOAc extract shows that the main ingredients were gigantol, moscatin, and dendrophenol. These ingredients (gigantol, moscatin, and dendrophenol) also showed more potent anti-AChE activity than chlorogenic acid (positive control) (Table 5). Therefore, we inferred that the potent anti-AChE activity of EtOAc extract was due to the presence of these three main active components: gigantol, moscatin, and dendrophenol. The phenanthrenoid moscatin (**2**) exhibited more potent anti-AChE activity than the 1,2-diphenylethane derivatives, dendrophenol (**4**), gigantol (**1**), and batatasin III (**3**). Among the 1,2-diphenylethane analogues (Table 5), gigantol (**1**) and dendrophenol (**4**) (with 4-hydroxy-3-methoxyphenethyl moiety) exhibited more potent anti-AChE activity than batatasin III (**3**) (with 3-hydroxyphenethyl moiety).

### 3.8. Anti-Inflammatory Activity

Anti-inflammatory effects were estimated by measuring the suppression of NO production in the RAW 264.7 cell line. As indicated in Table 6 and Table 7, among all solvent extracts, the *n*-hexane extract (IC_50_ = 9.19 ± 0.49 μg/mL) displayed the best inhibitory effect against NO production in vitro, followed by EtOAc and CH_2_Cl_2_ extracts (IC_50_ = 18.56 ± 1.54 and 20.60 ± 1.46 μg/mL, respectively). Among the isolated compounds, dendrophenol possessed the strongest anti-NO activity (IC_50_ = 14.31 ± 3.17 μM), followed by moscatin, batatasin III, and gigantol (IC_50_ = 23.46 ± 3.16, 51.32 ± 5.51, and 63.71 ± 11.41 μM, respectively). Notably, all crude extracts and most of the isolated compounds showed low cytotoxicity (Figures S3 and S4). HPLC analysis of EtOAc extract showed that the main ingredients were gigantol, moscatin, and dendrophenol. As for the component analysis of low-polarity *n*-hexane extract, further research is needed. Among the 1,2-diphenylethane analogues, dendrophenol (in the 4,4′-dihydroxy-3,3′,5′-trimethoxyl group) exhibited more effective anti-NO activity than its analogues, batatasin III (in the 3,3′-dihydroxy-5′-methoxyl group) and gigantol (in the 4,3′-dihydroxy-3,5′-dimethoxyl group). Therefore, our study suggests *D. fimbriatum* and its bioactive compounds (dendrophenol and moscatin) could be further discovered as promising candidates for the therapy or prevention of various inflammatory diseases.

### 3.9. Effects of Active Components on the Expression of iNOS

**Figure 3:**
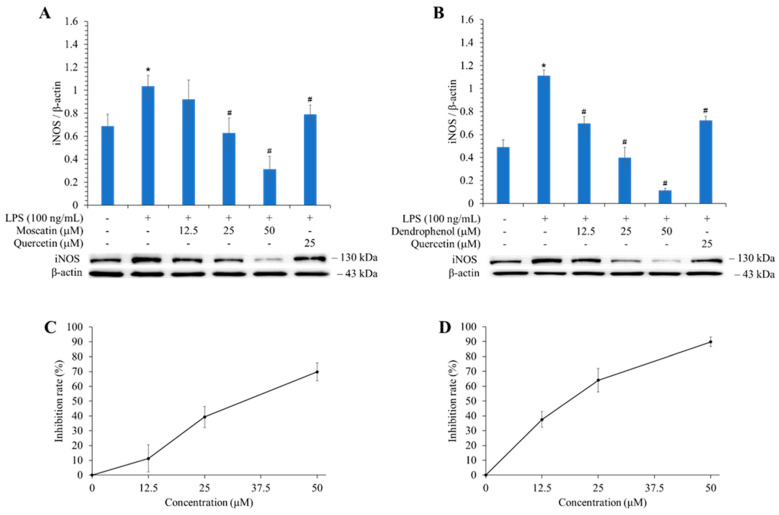
Inhibitory activities of moscatin and dendrophenol against LPS-induced iNOS expression in RAW 264.7 murine macrophages are assessed by Western blot. (**A**) The inhibitory effect of moscatin against LPS-induced iNOS in RAW 264.7 macrophage cell line. (**B**) The inhibitory activity of dendrophenol against LPS-induced iNOS in RAW 264.7 macrophage cell line. (**C**) The inhibition rate line chart of moscatin against LPS-induced iNOS in RAW 264.7 macrophage cell line. (**D**) The inhibition rate line chart of dendrophenol against LPS-induced iNOS in RAW 264.7 macrophage cell line. Quantification data of iNOS/β-actin are shown as mean ± SD (n = 3). Quercetin is applied as a positive control. * *p* < 0.05 compared with the control group, ^#^ *p* < 0.05 compared with the LPS group.

The anti-inflammatory effect was further conducted by evaluating the expression of iNOS in vitro, and the results are shown in Figure 3. The iNOS expression inhibitory activity of the two most potent compounds (dendrophenol and moscatin) was assessed by Western blotting. Dendrophenol inhibited iNOS expression by 89.82% at 50 μM, while moscatin suppressed 69.76% at the same concentration.

### 3.10. HPLC-ESI-qTOF-MS/MS Analysis

**Figure 4:**
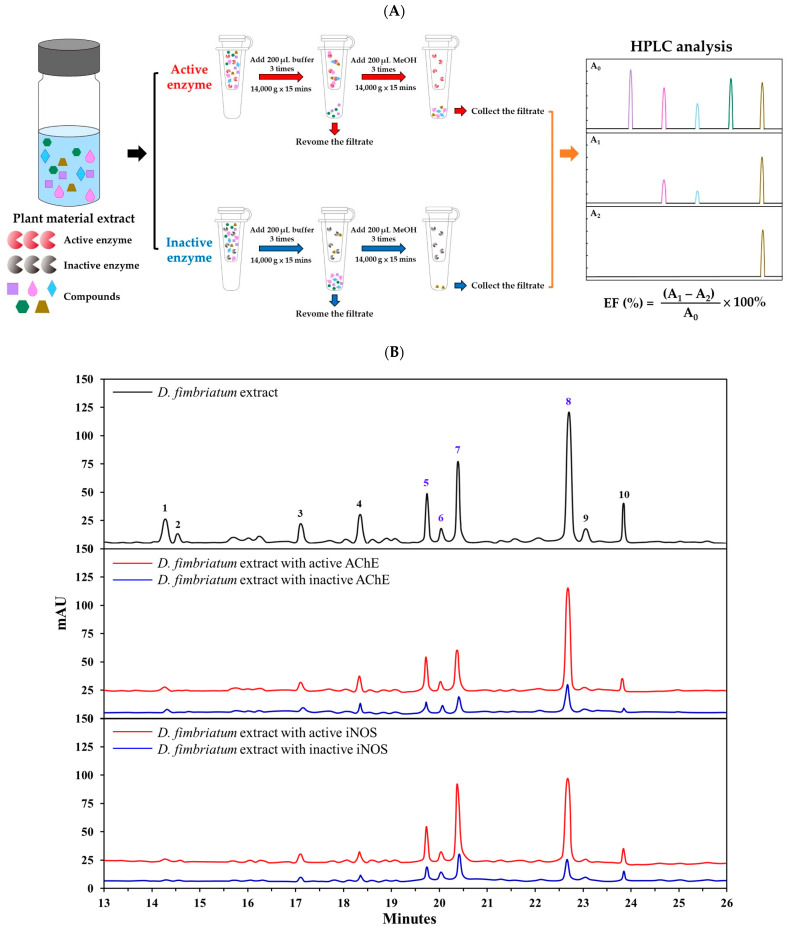
HPLC chromatograms of the potential AChE inhibitors in the EtOAc extract of *D. fimbriatum* stems obtained by bioaffinity ultrafiltration. (**A**) Schematic diagram of bioaffinity ultrafiltration assay. (**B**) HPLC chromatogram (280 nm) of the chemical components in the EtOAc extract of *D. fimbriatum* stem obtained by bioaffinity ultrafiltration. The black line represents *D. fimbriatum* stem extract without ultrafiltration, while the red and blue lines represent *D. fimbriatum* stem extract with active and inactive AChE and iNOS, respectively.

Ten components have been confirmed from the stem extracts of *D. fimbriatum.* The HPLC chromatogram at 280 nm for chemical components of the ethyl acetate extract is shown in Figure 4B. The HPLC-ESI-qTOF-MS/MS analysis information for main chemical components, including retention time, precursor ion, and MS/MS fragment ions, is listed in Table 8.

Peaks 1 to 10 were identified based on retention times (RT) and characteristic ions. Peak 1 (RT 14.2 min) was determined as 2,4,7-trihydroxy-9,10-dihydrophenanthrene by referring its MS fragment ion at *m*/*z* 151.07 and characteristic fragment ions at *m/z* 137.06 and 123.08 [C_14_H_12_O_3_ + H]^+^. Peak 2 (RT 14.5 min) was tentatively identified as dihydroresveratrol because of the existence of the parent ion at *m/z* 137.06 and characteristic fragment ions at *m/z* 121.07 and 107.05 [M + H]^+^. Peak 3 (RT 17.1 min), with the parent ions at *m/z* 153.06 [M + H]^+^ and characteristic fragment ion at *m*/*z* 129.06 and 116.06, was tentatively assigned as coelonin. Peak 4 (RT 18.4 min) was tentatively assigned as nudol, according to its parent ion at *m/z* 193.06 [C_16_H_14_O_4_ + H]^+^ and MS/MS fragment ions at *m/z* 181.06 and 165.07. Peak 5 (RT 19.7 min), with the precursor ion at *m/z* 137.06 [C_16_H_18_O_4_ + H]^+^ and MS/MS fragment ions at *m*/*z* 122.04 and 103.05, was temporarily assigned as gigantol. Peak 6, with the precursor ion at *m*/*z* 151.08 [C_15_H_16_O_3_ + H]^+^ and MS/MS fragment ions at *m*/*z* 121.06 and 105.04, was temporarily identified as batatasin III. Peak 7 indicated parent ion at *m*/*z* 181.09 [M + H]^+^ and characteristic fragment ions at *m*/*z* 151.08 and 137.06, and could be temporarily assigned as dendrophenol. Peak 8 (RT 22.7 min) was determined as moscatin by referring its parent ion at *m*/*z* 181.06 and MS fragment ion at *m*/*z* 152.06 and 127.05 [C_15_H_12_O_3_ + H]^+^. Peak 9 showed [M + H]^+^ parent ion at *m*/*z* 137.06 [C_15_H_16_O_4_ + H]^+^ and MS fragment ions at *m*/*z* 122.04 and 107.05, which could be determined as erianin. Peak 10 (RT 23.8 min) was determined as tristin, considering the parent ion at *m*/*z* of 129.07 [M + H]^+^ and fragment ions at *m*/*z* 128.06 and 115.05.

### 3.11. Bioaffinity Ultrafiltration

Because the ethyl acetate extract of *D. fimbriatum* stems exhibited potent AChE and NO inhibitory activity, further studies were performed using the bioaffinity ultrafiltration method, and the bound ligands in the ethyl acetate extract of *D. fimbriatum* stems were released by methanol solution and analyzed by HPLC-ESI-qTOF-MS/MS to identify AChE and iNOS inhibitory components. The binding affinity of compounds to the enzyme was determined using the enrichment factor (EF). The binding affinities of four major components were evaluated and the results are shown in Table 8 and Figure 4.

Moscatin, dendrophenol, gigantol, and batatasin III incubated with AChE in the ethyl acetate extract from the stems of *D. fimbriatum* showed higher bioaffinity ability when compared with the inactivated control group, with affinity values of 66.31%, 59.48%, 54.60%, and 31.87%, respectively. These results indicated that these four constituents showed specific binding toward AChE. Therefore, these four constituents were considered major potential AChE ligands.

As presented in Table 8, the affinity capacity of the identified compounds with iNOS can be ranked as moscatin (88.99%) > dendrophenol (65.11%) > gigantol (44.84%) > batatasin III (27.18%) > nudol (24.39%) > coelonin (5.09%). Among the components of the EtOAc extract of *D. fimbriatum*, moscatin had the highest binding affinity for iNOS. The analysis results further confirmed that the main active compound (moscatin) can effectively inhibit the expression of iNOS. Furthermore, among the phenanthrenoid analogues (moscatin and nudol) (Figure S5), moscatin (in the 2,5-dihydroxy-4-methoxy group) exhibited higher iNOS affinity than its analogue nudol (in the 4,7-dihydroxy-2-methoxy group).

### 3.12. Molecular Docking Analysis

**Figure 5:**
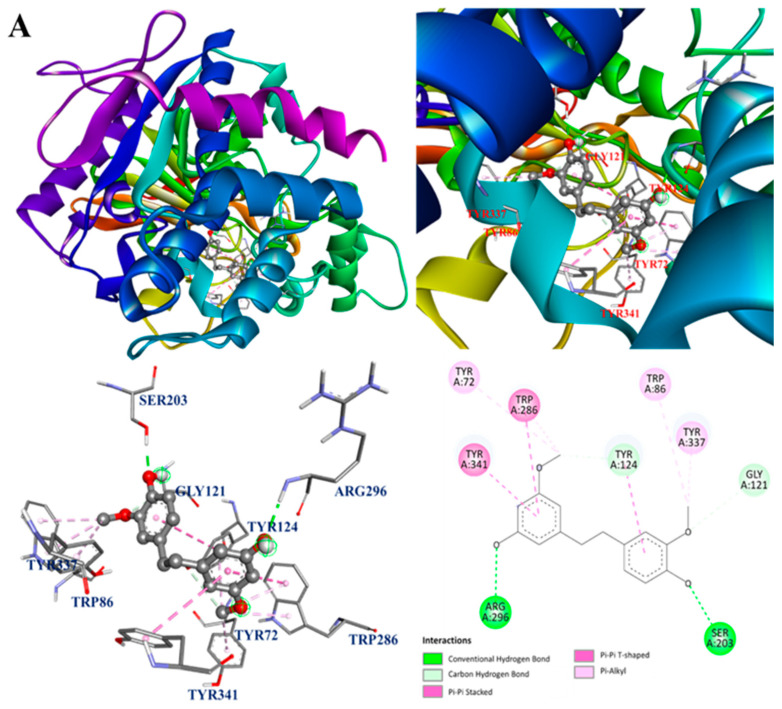

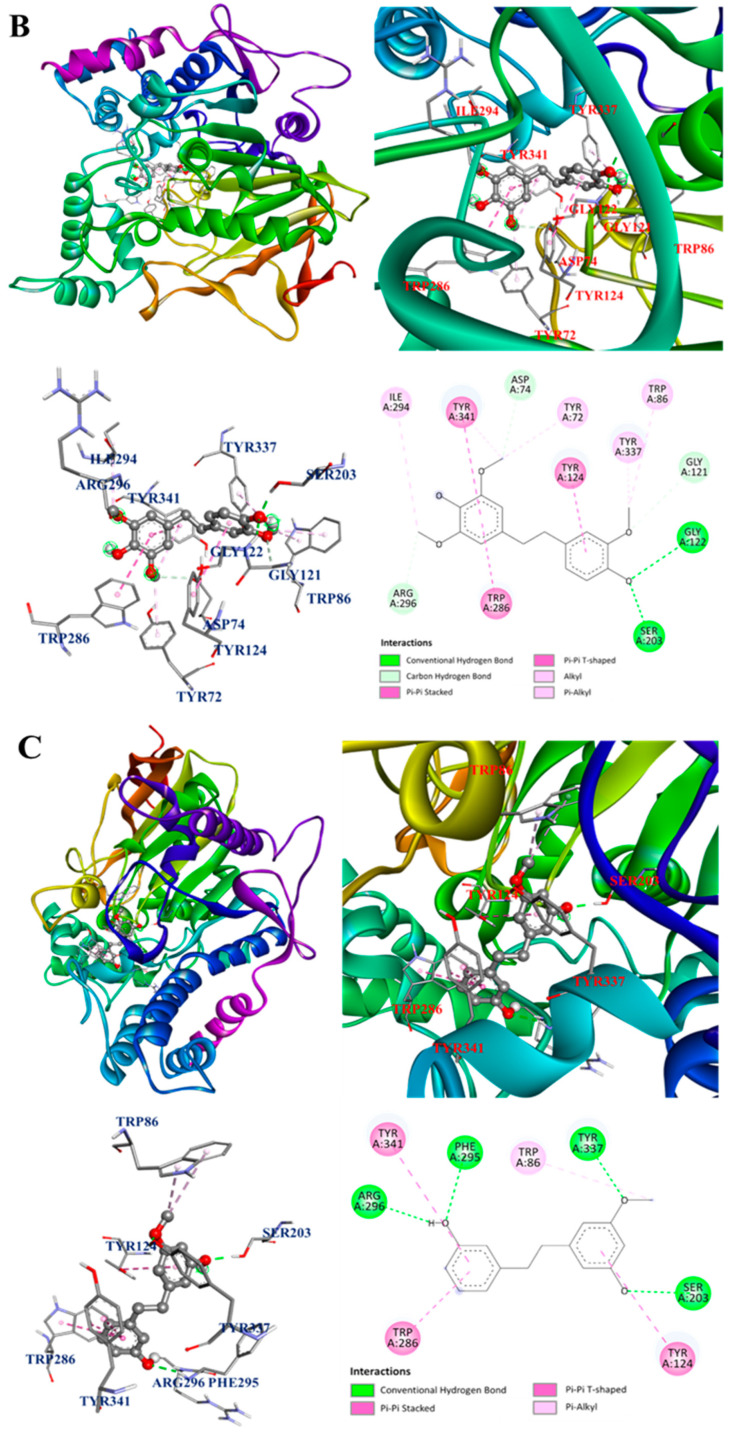

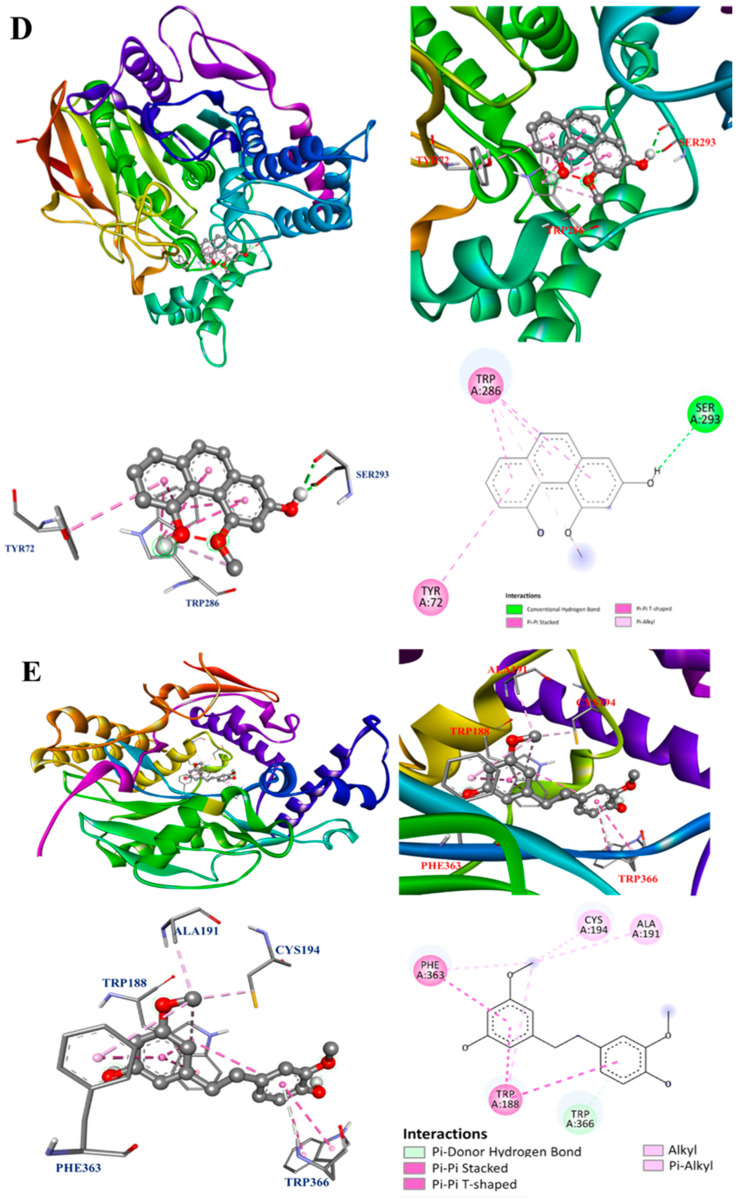

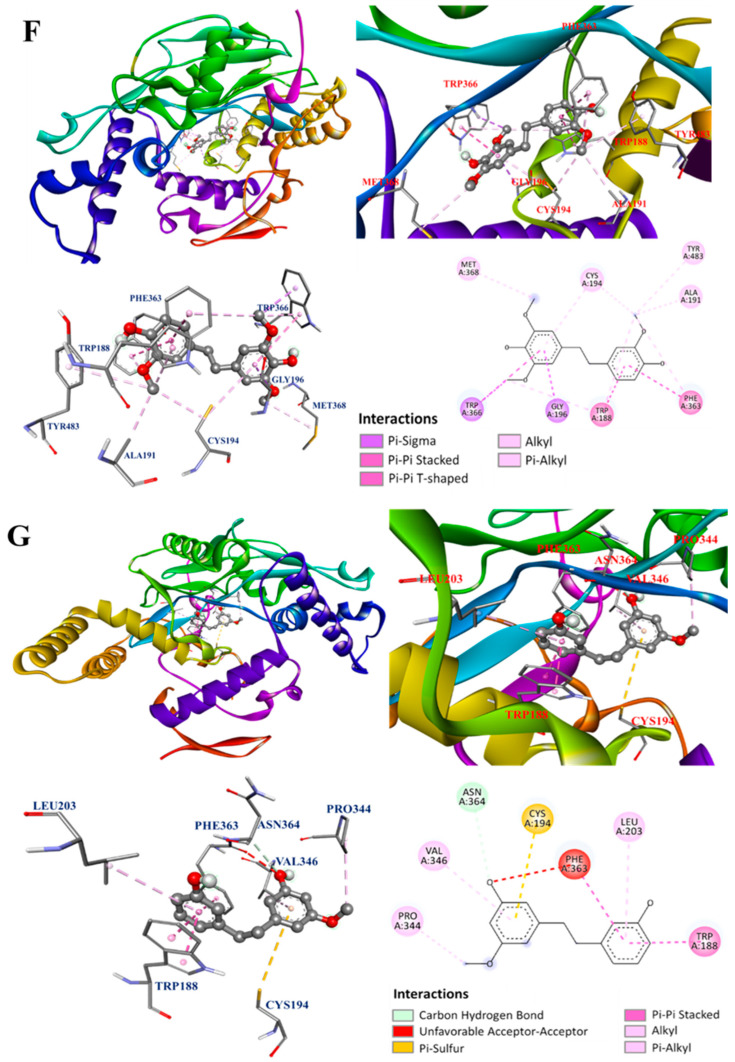

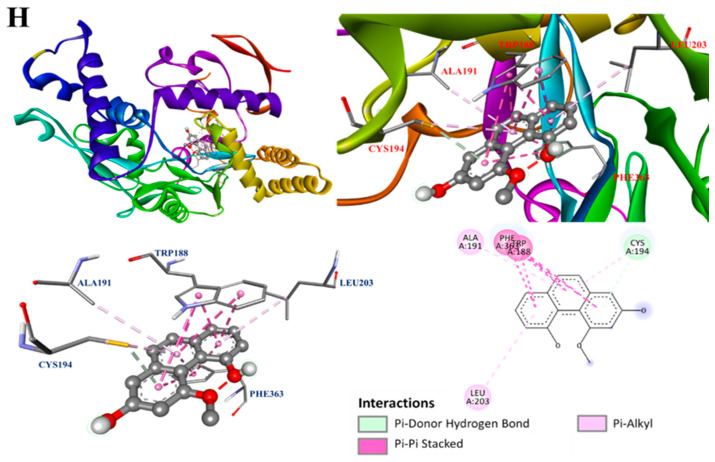
(**A**) Interaction of gigantol with the active sites of *E. electric* AChE. (**B**) Interaction of dendrophenol with the active sites of *E. electric* AChE. (**C**) Interaction of batatasin III with the active sites of *E. electric* AChE. (**D**) Interaction of moscatin with the active sites of *E. electric* AChE. (**E**) Interaction of gigantol with the active sites of *M. musculus* iNOS. (**F**) Interaction of dendrophenol with the active sites of *M. musculus* iNOS. (**G**) Interaction of batatasin III with the active sites of *M. musculus* iNOS. (**H**) Interaction of moscatin with the active sites of *M. musculus* iNOS.

Since the main bioactive components of *D. fimbriatum* showed potent inhibitory activity against AChE and iNOS, further molecular docking analysis was carried out. The interactions between the bioactive components of *D. fimbriatum* and AChE and iNOS are shown in Figure 5A–H, and the molecular docking model of gigantol, dendrophenol, batatasin III, moscatin, and AChE is shown in Figure 5A–D. The interactions between active compounds and PDB: 1C2B were displayed in the best docked poses for the calculations. According to the results of the acetylcholinesterase inhibitory effect test (Table 5) and binding energy results (Table 8), moscatin had anti-AChE effect potential. Moscatin (Figure 5D) formed a conventional hydrogen bond with Ser293 of AChE and bound to Tyr72 of AChE via π-π T-shaped interactions. Additionally, π-π stacked interactions and π-alkyl interactions were found with Trp286 of AChE. Interestingly, gigantol (**1**) and moscatin (**2**) were both bound to acetylcholinesterase, exhibiting the same binding energies (BE = −8.6 kcal/mol) (Table 8). However, as shown in Table 5, moscatin (IC_50_ = 161.86 ± 16.45 μM) demonstrated better anti-AChE activity than gigantol (IC_50_ = 176.05 ± 19.08 μM). Therefore, binding energy (Table 8) was the calculated estimated value of molecular docking, which may sometimes be slightly different from the experimental value. Furthermore, according to bioaffinity ultrafiltration and HPLC analysis (Figure 4), moscatin (66.31%) exhibited higher binding affinity (EF) to acetylcholinesterase than gigantol (54.60%) (Table 8).

The interactions between active compounds and PDB: 1M9T (the 3D structure of iNOS from *Mus musculus* was used as a docking model) were displayed in the best-docked poses for calculations. The active site of PDB: 1M9T comprised four pockets, including the substrate-binding S pocket containing heme [33]. The molecular docking model of gigantol, dendrophenol, batatasin III, moscatin, and iNOS is shown in Figure 5E–H.

Based on the results of the NO production inhibition test (Table 7) and the Western blotting results of the related protein, inducible nitric oxide synthase (iNOS) (Figure 3), both active compounds (moscatin and dendrophenol) exhibited anti-inflammatory potential. As shown in Figure 5F, the compound was dendrophenol, which bound to iNOS through π-sigma interactions with Trp366 and Gly196 and π-alkyl interactions with Ala191, Cys194, Tyr483, and Met368.

The interactions between moscatin and iNOS are shown in Figure 5H. Moscatin bound to iNOS through π-π stacked interactions with Trp188 and Phe363 and π-alkyl interactions with Ala191, Cys194, and Leu203. Furthermore, a π-donor hydrogen bond was found between moscatin and Cys194 of iNOS.

The molecular docking analysis results (Figure 5A–H) of other isolated compounds and AChE and iNOS also showed that these active compounds displayed good affinity to AChE and iNOS, which was consistent with previous activity test data.

## 4. Conclusions

Eight solvent extracts of *D. fimbriatum* stems were evaluated for their antioxidant, anti-inflammatory, anti-α-glucosidase, and anti-AChE activities and TPC, TFC, and yield. Among all extracts, EtOAc and acetone extracts exhibited the highest TPC, and their antioxidant activities also displayed the same trend. The TFC of various solvent extracts decreased with the increase in polarity of the extraction solvent. Ethyl acetate and acetone extracts exhibited the strongest antioxidant effect in FRAP, ABTS, and DPPH radical scavenging tests. The acetone extract revealed the most potent inhibitory activity against α-glucosidase, while the EtOAc extract possessed the best inhibitory activity against AChE. The *n*-hexane extract exhibited the strongest anti-inflammatory activities against NO production in vitro.

Among the isolated components, gigantol, moscatin, and dendrophenol exhibited potent antioxidant effects in FRAP, ABTS, and DPPH radical scavenging tests. Dendrophenol also exhibited the best inhibitory activity (IC_50_ = 14.31 ± 3.17 μM) against NO production and was more potent than quercetin (positive control, IC_50_ = 23.09 ± 1.43 μM). Dendrophenol and moscatin (50 μM) inhibited iNOS expression by 89.82% and 69.76%, respectively, by Western blot analysis. Gigantol (IC_50_ = 469.72 ± 28.51 μM) and moscatin (IC_50_ = 478.46 ± 33.43 μM) revealed more efficient anti-α-glucosidase function than acarbose (positive control, IC_50_ = 490.00 ± 17.23 μM), while moscatin (IC_50_ = 161.86 ± 16.45 μM) and dendrophenol (IC_50_ = 165.19 ± 13.25 μM) exhibited more potent anti-AChE activity than chlorogenic acid (positive control, IC_50_ = 236.24 ± 15.85 μM). In the bioaffinity ultrafiltration experiment and molecular docking analysis, the results further confirmed that these active compounds (dendrophenol, moscatin, and/or gigantol) had good affinity with related enzymes (AChE and/or iNOS), which was consistent with actual test data.

In conclusion, our study demonstrated that the bioactive extracts and components of *D. fimbriatum* stem are excellent natural anti-inflammatory and antioxidant agents, and potent inhibitors of acetylcholinesterase and α-glucosidase. Since AChE and α-glucosidase play crucial roles in the pathogenesis of Alzheimer’s disease and diabetes mellitus (DM), the bioactive extracts and components (especially dendrophenol, moscatin, and gigantol) of *D. fimbriatum* stem can be used as health supplements to prevent and improve Alzheimer’s disease and DM.

## Figures and Tables

**Table 1:** TPC, TFC, and extraction yields of *Dendrobium fimbriatum* with each extraction solvent.

Extracting Solvents	TPC (mg/g) ^A^ (GAE)	TFC (mg/g) ^B^ (QE)	Yields (%) ^C^
*n*-Hexane	1.67 ± 0.27	51.53 ± 1.88	0.30 ± 0.08
Dichloromethane	18.17 ± 2.48	49.69 ± 1.96	0.74 ± 0.05
Ethyl acetate	33.92 ± 0.75	44.05 ± 0.65	0.70 ± 0.04
Acetone	34.48 ± 1.50	34.20 ± 3.43	1.10 ± 0.03
Methanol	5.88 ± 0.16	<1.00	7.46 ± 0.12
Ethanol	9.91 ± 0.34	3.46 ± 0.26	14.11 ± 0.37
Water	3.43 ± 0.09	<1.00	11.40 ± 0.28
Water 100 °C	5.54 ± 0.24	<1.00	19.70 ± 1.64

^A^ TPC is expressed as mg of gallic acid equivalents (GAE) per g of extract; ^B^ TFC is expressed as mg of quercetin equivalents (QE) per g of extract; ^C^ extraction yield is calculated as yields (%) = (weight of extract/initial weight of dry sample) × 100; values are expressed as means ± SD (n = 3).

**Table 2:** Antioxidant activities of different solvent extracts from *Dendrobium fimbriatum* determined by DPPH, ABTS, superoxide radical scavenging, and FRAP assays.

Extracting Solvents	SC_50_ (μg/mL) ^A^			TE (mM/g) ^C^
	DPPH	ABTS	Superoxide	FRAP
*n*-Hexane	>400	>400	>400	10.78 ± 0.69 ^c^
Dichloromethane	88.44 ± 13.19 ^b^	108.47 ± 9.03 ^b^	>400	562.26 ± 14.21 ^c^
Ethyl acetate	53.08 ± 8.37 ^b^	57.62 ± 5.38 ^b^	>400	882.70 ± 7.26 ^c^
Acetone	55.89 ± 7.68 ^b^	59.78 ± 5.26 ^b^	>400	861.19 ± 11.31 ^a^
Methanol	>400	>400	>400	162.15 ± 7.59 ^c^
Ethanol	374.61 ± 27.50 ^c^	>400	>400	273.21 ± 7.98 ^c^
Water	>400	>400	>400	85.29 ± 3.04 ^c^
Water 100 °C	>400	>400	>400	133.19 ± 2.73 ^c^
BHT ^B^	36.64 ± 2.22	46.83 ± 0.44	-	4296.98 ± 48.40
Cynaroside ^B^	-	-	112.76 ± 5.84	-

^A^ The SC_50_ value is defined as the concentration of the samples causing 50% free radical scavenging and is displayed as mean ± SD (n = 3); ^B^ butylated hydroxytoluene (BHT) and cynaroside are applied as positive controls; ^C^ FRAP is expressed as millimolar (mM) of Trolox equivalents (TE) per g of extract; ^a^ *p* < 0.05, ^b^ *p* < 0.01, and ^c^ *p* < 0.001 compared with the positive control.

**Table 3:** Antioxidant activities of isolated components from *Dendrobium fimbriatum* determined by DPPH, ABTS, and superoxide radical scavenging, and FRAP assays.

Compounds	SC_50_ (μM) ^A^			TE (mM/g) ^C^
	DPPH	ABTS	Superoxide	FRAP
Gigantol (**1**)	35.18 ± 1.34 ^c^	12.25 ± 0.54 ^c^	>400	1483.20 ± 63.49 ^c^
Moscatin (**2**)	26.66 ± 1.63 ^c^	10.34 ± 1.92 ^c^	>400	1396.40 ± 57.03 ^c^
Batatasin III (**3**)	>400	30.14 ± 1.70 ^b^	>400	559.67 ± 51.49 ^c^
Dendrophenol (**4**)	11.64 ± 0.77 ^c^	7.64 ± 0.63 ^c^	>400	1686.53 ± 16.61 ^c^
BHT ^B^	90.77 ± 5.65	53.72 ± 1.48	-	4355.95 ± 99.23
Cynaroside ^B^	-	-	50.56 ± 2.62	-

^A^ The SC_50_ value is defined as the concentration of the samples causing 50% free radical scavenging and is displayed as mean ± SD (n = 3); ^B^ butylated hydroxytoluene (BHT) and cynaroside are applied as positive controls; ^C^ FRAP is expressed as millimolar (mM) of Trolox equivalents (TE) per g of extract; ^b^ *p* < 0.01 and ^c^ *p* < 0.001 compared with the positive control.

**Table 4:** Anti-α-glucosidase and acetylcholinesterase inhibitory effects of different solvent extracts.

Extracting Solvents	IC_50_ (μg/mL) ^A^	
	*α*-Glucosidase	AChE
*n*-Hexane	91.43 ± 2.87 ^c^	13.77 ± 4.09 ^c^
Dichloromethane	294.09 ± 8.61	31.89 ± 6.89 ^b^
Ethyl acetate	62.02 ± 0.21 ^c^	11.59 ± 1.52 ^b^
Acetone	51.62 ± 0.84 ^c^	92.05 ± 5.58 ^a^
Methanol	>400	80.71 ± 8.44
Ethanol	>400	51.96 ± 4.92 ^a^
Water	>400	133.53 ± 8.04 ^b^
Water 100 °C	>400	75.87 ± 7.91
Acarbose ^B^	316.30 ± 11.15	-
Chlorogenic acid ^B^	-	75.99 ± 6.21

^A^ The IC_50_ value is defined as half-maximal inhibitory concentration and is expressed as mean ± SD (n = 3); ^B^ acarbose and chlorogenic acid are used as positive controls for anti-α-glucosidase and anti-AChE assays, respectively; ^a^ *p* < 0.05, ^b^ *p* < 0.01, and ^c^ *p* < 0.001 compared with the positive control.

**Table 5:** Anti-α-glucosidase and acetylcholinesterase inhibitory effects of isolated compounds.

Compounds	IC_50_ (μM) ^A^	
	*α*-Glucosidase	AChE
Gigantol (**1**)	469.72 ± 28.51	176.05 ± 19.08 ^b^
Moscatin (**2**)	478.46 ± 33.43	161.86 ± 16.45 ^b^
Batatasin III (**3**)	723.21 ± 53.54 ^c^	258.29 ± 18.52
Dendrophenol (**4**)	608.37 ± 48.16 ^c^	165.19 ± 13.25 ^c^
Acarbose ^B^	490.00 ± 17.23	-
Chlorogenic acid ^B^	-	236.24 ± 15.85

^A^ The IC_50_ value is defined as half-maximal inhibitory concentration and is expressed as mean ± SD (n = 3); ^B^ acarbose and chlorogenic acid are used as positive controls for anti-α-glucosidase and anti-AChE assays, respectively; ^b^ *p* < 0.01 and ^c^ *p* < 0.001 compared with the positive control.

**Table 6:** Effects of different solvent extracts on nitric oxide (NO) production in RAW 264.7 cells.

Extracting Solvents	NO Inhibition IC_50_ (μg/mL) ^A^
*n*-Hexane	9.19 ± 0.49 ^b^
Dichloromethane	20.60 ± 1.46 ^b^
Ethyl acetate	18.56 ± 1.54 ^b^
Acetone	21.67 ± 1.05 ^c^
Methanol	66.48 ± 1.42 ^c^
Ethanol	22.55 ± 1.28 ^b^
Water	33.49 ± 1.04 ^c^
Water 100 °C	25.24 ± 0.24 ^c^
Quercetin ^B^	6.98 ± 0.43

^A^ The IC_50_ value is defined as half-maximal inhibitory concentration and is expressed as mean ± SD (n = 3); ^B^ quercetin is used as a positive control; ^b^ *p* < 0.01 and ^c^ *p* < 0.001 compared with the positive control.

**Table 7:** Effects of isolated components on nitric oxide (NO) production in RAW 264.7 cells.

Compounds	NO Inhibition IC_50_ (μM) ^A^
Gigantol (**1**)	63.71 ± 11.41 ^a^
Moscatin (**2**)	23.46 ± 3.16
Batatasin III (**3**)	51.32 ± 5.51 ^a^
Dendrophenol (**4**)	14.31 ± 3.17 ^a^
Quercetin ^B^	23.09 ± 1.43

^A^ The IC_50_ value is defined as half-maximal inhibitory concentration and is expressed as mean ± SD (n = 3); ^B^ quercetin is used as a positive control; ^a^ *p* < 0.05 compared with the positive control.

**Table 8:** Identification of main chemical compositions from the EtOAc extract of stems of *Dendrobium fimbriatum* using HPLC-ESI-qTOF-MS/MS and in silico analysis of bioactive compounds with acetylcholinesterase (AChE) and iNOS.

No.	RT ^a^ (min)	[M + H]^+^	MS/MS (*m*/*z*)	Mw	Compounds	AChE ^b^			iNOS ^c^		
						EF (%) ^d^	BE (kcal/mol) ^e^	Active Amino Acid Residues	EF (%) ^d^	BE (kcal/mol) ^e^	Active Amino Acid Residues
1	14.2	229.08	151.07 137.06 123.08	C_14_H_12_O_3_	2,4,7-Trihydroxy-9,10-dihydrophenanthrene	1.32	–	–	0.91	–	–
2	14.5	231.10	137.06 121.07 107.05	C_14_H_14_O_3_	Dihydroresveratrol	0.08	–	–	0.62	–	–
3	17.1	243.10	153.06 129.06 116.06	C_15_H_14_O_3_	Coelonin	2.00	–	–	5.09	–	–
4	18.4	271.10	193.06 181.06 165.07	C_16_H_14_O_4_	Nudol	29.56	–	–	24.39	–	–
5	19.7	275.13	137.06 122.04 103.05	C_16_H_18_O_4_	Gigantol	54.60	−8.6	Arg296, Gly121, Ser203, Trp86, Trp286, Tyr72, Tyr124, Tyr337, Tyr341	44.84	−7.9	Ala191, Cys194, Phe363, Trp188, Trp366
6	20.0	245.12	151.08 121.06 105.04	C_15_H_16_O_3_	Batatasin III	31.87	−8.5	Arg296, Phe295, Ser203, Trp86, Trp286, Tyr124, Tyr337, Tyr341	27.18	−8.2	Asn364, Cys194, Leu203, Phe363, Pro344, Trp188, Val346
7	20.4	305.14	181.09 151.08 137.06	C_17_H_20_O_5_	Dendrophenol	59.48	−8.0	Arg296, Asp74, Gly121, Gly122, Ile294, Ser203, Trp86, Trp286, Tyr72, Tyr124, Tyr337, Tyr341	65.11	−7.8	Ala191, Cys194, Gly196, Met368, Phe363, Trp188, Trp366, Tyr483
8	22.7	241.09	181.06 152.06 127.05	C_15_H_12_O_3_	Moscatin	66.31	−8.6	Ser293, Trp286, Tyr72	88.99	−9.1	Ala191, Cys194, Leu203, Phe363, Trp188
9	23.0	319.15	137.06 122.04 107.05	C_18_H_22_O_5_	Erianin	0.23	–	–	1.72	–	–
10	23.8	261.11	129.07 128.06 115.05	C_15_H_16_O_4_	Tristin	5.83	–	–	0.95	–	–

^a^ RT—retention time; ^b^ AChE—acetylcholinesterase; ^c^ iNOS—inducible nitric oxide synthase; ^d^ EF—enrichment factor; ^e^ BE—binding energy.

## Data Availability

Data are contained within the article and Supplementary Materials.

## Supplementary Materials

The following supporting information can be downloaded at: https://www.mdpi.com/article/10.3390/antiox13080918/s1. Figure S1: Gallic acid calibration lines in the concentration range 0–30 μg/mL. Figure S2: Quercetin calibration lines in the concentration range 0–80 μg/mL. Figure S3: Evaluation of cell viability of solvent extracts of *D. fimbriatum* by LPS-induced RAW 264.7. Figure S4: Evaluation of cell viability of pure compounds of *D. fimbriatum* by LPS-induced RAW 264.7. Figure S5: Chemical structures of moscatin and nudol from *D. fimbriatum*.
