# Supplementary material for: Bioaffinity Ultrafiltration Combined with HPLC-ESI-qTOF-MS/MS for Screening Potential Bioactive Components from the Stems of Dendrobium fimbriatum and In Silico Analysis
Source: Antioxidants (Basel). 2024 Jul 29;13(8):918. doi: 10.3390/antiox13080918 (PMC11351458; doi:10.3390/antiox13080918)
Supplement: Supplementary file 1 [file antioxidants-13-00918-s001.zip › antioxidants-3091502-supplementary.pdf]

Supplementary data

Bioaffinity Ultrafiltration Combined with HPLC-ESI-qTOF-MS/MS for Screening Potential Bioactive Components from the Stems of *Dendrobium fimbriatum* and *in Silico* Analysis

Yu-Hui Hsieh [1], Wu-Chang Chuang [2,*], Ming-Chung Lee [3], Yu-Hsin Fan [4], Nai-Kuei Huang [5,6,7,*], and Jih-Jung Chen [1,4,8,9,*]

1 Biomedical Industry Ph.D. Program, School of Life Sciences, National Yang Ming Chiao Tung University, Taipei 112304, Taiwan; hsieh.ls10@nycu.edu.tw (Y.-H.H.)

2 Brion Research Institute of Taiwan, New Taipei City 231030, Taiwan; cwctd331@sunten.com.tw (W.-C.C.)

3 Sun Ten Pharmaceutical Co., Ltd., New Taipei City 231030, Taiwan; mileslee@sunten.com.tw (M.-C.L.)

4 Department of Pharmacy, School of Pharmaceutical Sciences, National Yang Ming Chiao Tung University, Taipei 112304, Taiwan; cindyfan.md12@nycu.edu.tw (Y.-H.F.)

5 National Research Institute of Chinese Medicine, Ministry of Health and Welfare, Taipei, 112304, Taiwan

6 Ph.D. Program for Neural Regenerative Medicine, College of Medical Science and Technology, Taipei Medical University, Taipei, 110301, Taiwan

7 Graduate Institute of Medical Sciences, College of Medicine, Taipei Medical University, Taipei, 110301, Taiwan

8 Department of Medical Research, China Medical University Hospital, China Medical University, Taichung 404333, Taiwan

9 Traditional Herbal Medicine Research Center, Taipei Medical University Hospital, Taipei 110301, Taiwan

---

* Corresponding author.

E-mail addresss: jjungchen@nycu.edu.tw (J.-J. Chen); cwctd331@sunten.com.tw (W.-C.C.); andrew@nricm.edu.tw (N.-K. Huang)

Contents

Figure S1. Gallic acid calibration lines in the concentration range 0–30 μg/mL…………….. S3

Figure S2. Quercetin calibration lines in the concentration range 0–80 μg/mL…………….. S3

Figure S3. Evaluation of cell viability of solvent extracts of *D. fimbriatum* by LPS-induced RAW 264.7. .... S4

Figure S4. Evaluation of cell viability of pure compounds of *D. fimbriatum* by LPS-induced RAW 264.7…………………….. S4

Figure S5. Chemical structures of moscatin and nudol from *D. fimbriatum*............................. S5

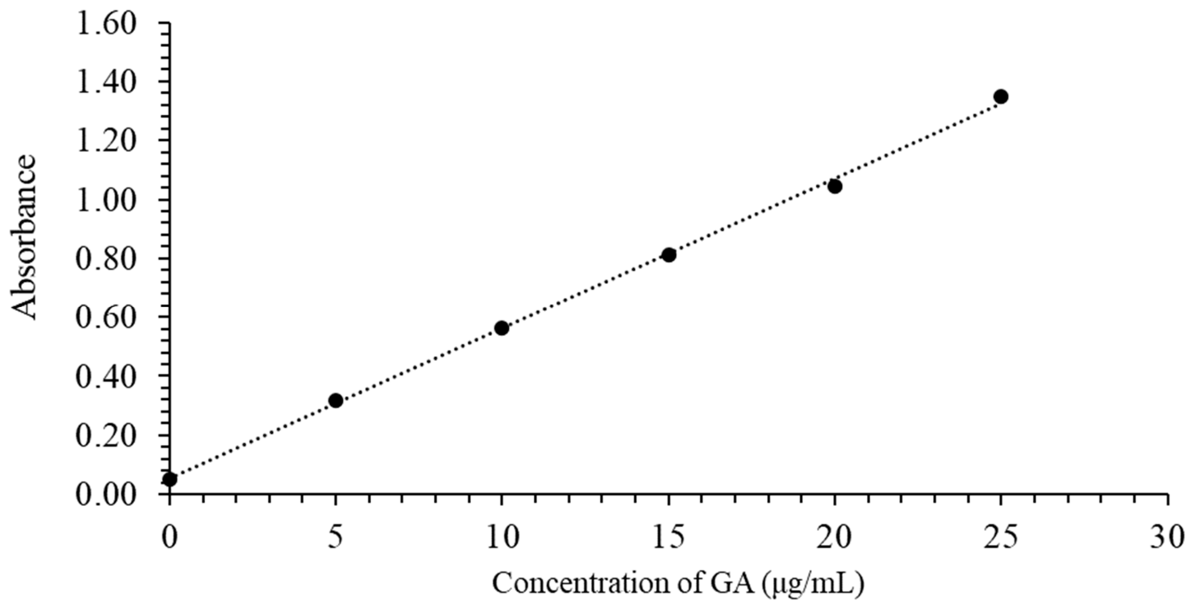

Figure S1. Gallic acid calibration lines in the concentration range 0–30 μg/mL. The dotted line corresponds to the equation: y = 0.051x + 0.0526, R^2 = 0.9989. The results calculated using this equation were converted into units of mg/g and presented in Table 1.

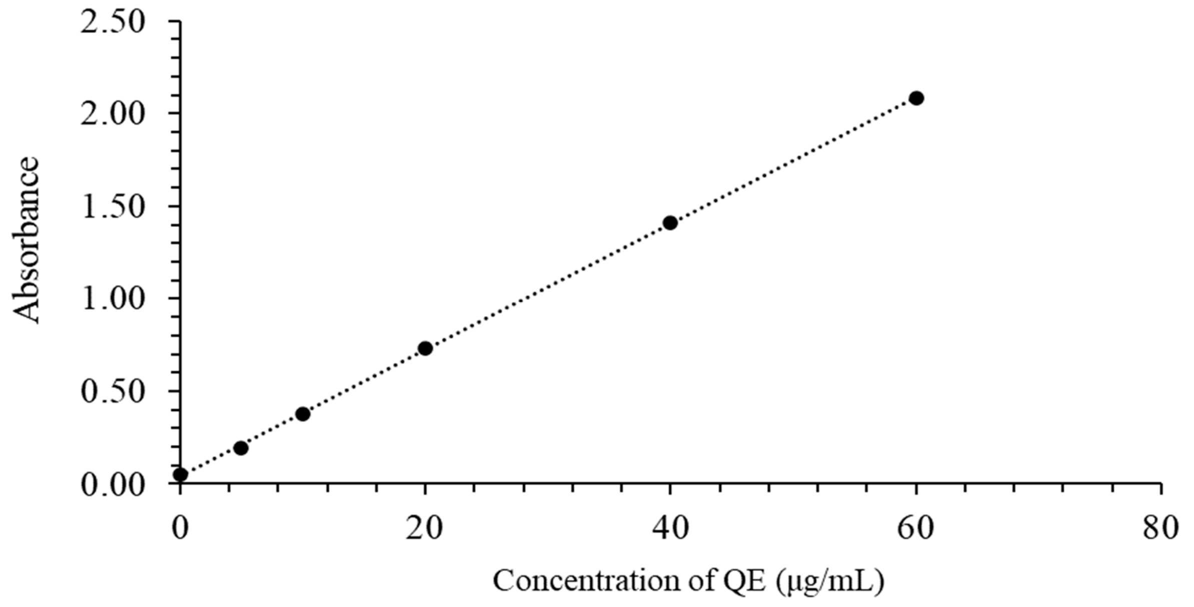

Figure S2. Quercetin calibration lines in the concentration range 0–80 μg/mL. The dotted line corresponds to the equation: y = 0.0326x + 0.0675, R^2 = 0.9985. The results calculated using this equation were converted into units of mg/g and presented in Table 1.

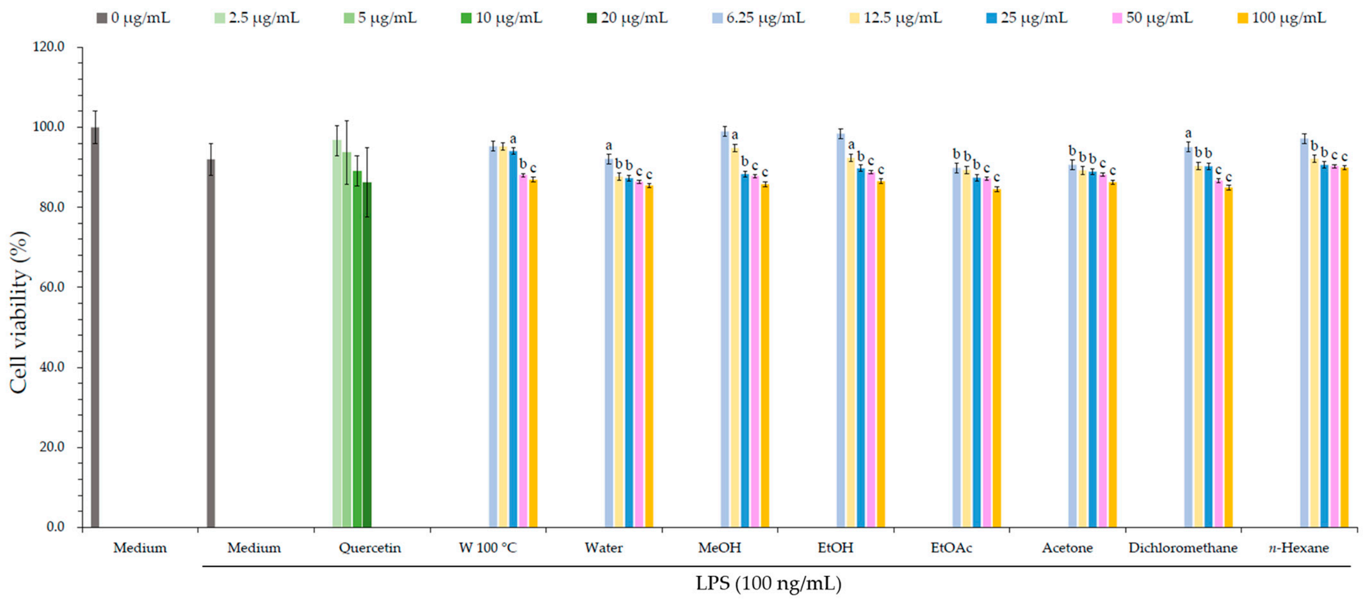

Figure S3. Cell viability of different solvent extracts of *D. fimbriatum* by LPS-induced RAW 264.7. Quercetin was used as a positive control. Data are displayed as mean ± SD (n = 3); ^{a} $p < 0.05$, ^{b} $p < 0.01$, and ^{c} $p < 0.001$ compared with the vehicle (Medium + LPS).

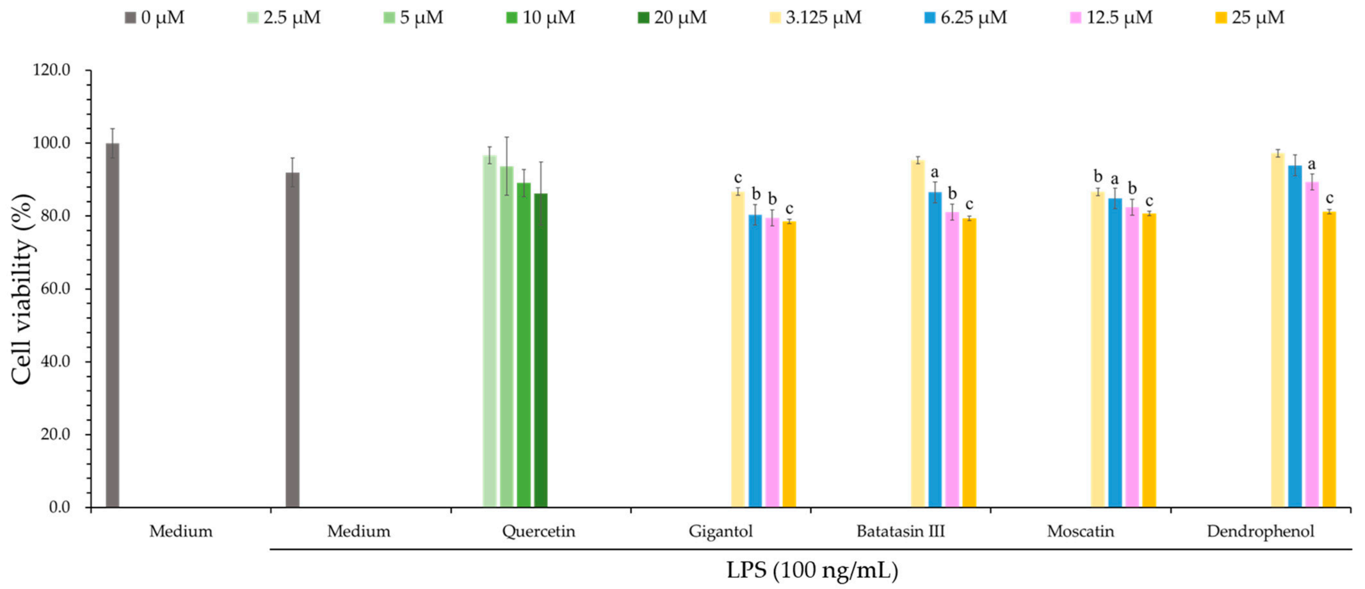

Figure S4. Cell viability of pure compounds of *D. fimbriatum* by LPS-induced RAW 264.7. Quercetin was used as a positive control. Data are displayed as mean ± SD (n = 3); ^{a} $p < 0.05$, ^{b} $p < 0.01$, and ^{c} $p < 0.001$ compared with the vehicle (Medium + LPS).

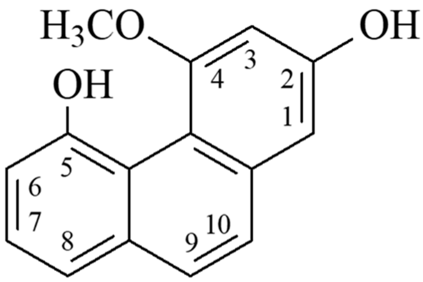

Moscatin

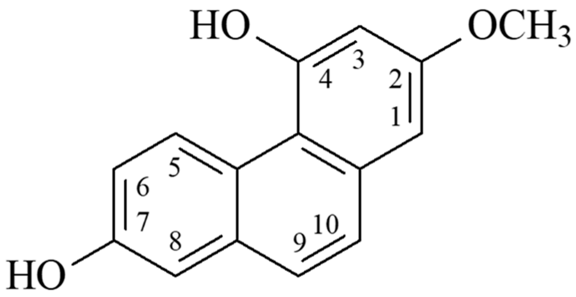

Nudol

Figure S5. Chemical structures of moscatin and nudol from *D. fimbriatum*.
